# How I do it: surgical resection of micro-arteriovenous malformations

**DOI:** 10.1007/s00701-025-06455-1

**Published:** 2025-02-13

**Authors:** Sergio Corvino, A. Yohan Alexander, Giuseppe Lanzino

**Affiliations:** 1 Department of Neurological Surgery, Mayo Clinic, 200 First St. SW Floor 8, Rochester, MN 55905 USA; 2 Department of Neurosciences, Reproductive and Odontostomatological Sciences, University of Naples Federico II, via S. Pansini 5, Naples, 80131 Italy; 3 Medical School, University of Minnesota, Minneapolis, MN USA; 4 Department of Radiology, Mayo Clinic, Rochester, MN USA

**Keywords:** Micro-arteriovenous malformations, Vascular malformations, Microsurgery, Surgical techniques, Intracranial vascular lesion

## Abstract

**Background:**

Micro-arteriovenous malformations (AVMs) are a subgroup of AVMs with a nidus smaller than 1- cm. As such, intraoperative localization of micro-AVMs can be challenging. Once identified intraoperatively, however, treatment of the micro-AVM is often straightforward.

**Method:**

We discuss and illustrate the fundamental imaging and intraoperative steps to localize micro-AVMs with the aid of an illustrative clinical case.

**Conclusion:**

Micro-AVMs can be challenging to localize intraoperatively. With the aid of anatomical, clinical, and intraoperative findings, the micro-AVMs small draining vein can be localized and traced in a retrograde fashion to identify and remove the micro-AVM.

**Supplementary Information:**

The online version contains supplementary material available at 10.1007/s00701-025-06455-1.

## Manuscript

### Relevant surgical anatomy

Micro-arteriovenous malformations (AVMs), as originally described by Yasargil, are AVMs with a nidus smaller than 1 cm [9]. Like AVMs in general, micro-AVMs are a tangle of arteriovenous connections without a capillary bed. Consequently, in theory micro-AVMs can occur anywhere in the central nervous system. Most reported micro-AVMs, however, occur in the supratentorial space [1]. More specifically, they may be more likely to occur in anatomical locations with a high concentration of draining veins. Regarding diagnosis, the vast majority of micro-AVMs are diagnosed after intraparenchymal hemorrhage [1, 5]. Given the very small size and relatively low flow of micro-AVMs, it is not uncommon that the first diagnostic catheter angiogram is not revealing as the micro-AVM can be easily compressed by the pressure of the fresh hemorrhage [1]. Many true micro-AVMs are diagnosed on a repeat catheter angiogram which we usually consider 6–12 weeks after the initial bleeding episode. Intraoperatively, one can expect to find the micro-nidus of the micro-AVM consistently along the border of the hemorrhage cavity.

### Description of the technique

Given the very small size, proper localization of the micro-nidus and its feeding arteries and draining vein/veins is paramount and often constitutes the most challenging portion of the procedure. The craniotomy is fashioned with the aid of neuro-navigation based on computed tomography (CT) or magnetic resonance (MR) angiography. As the AVM’s micro-nidus may not be readily apparent on non-invasive vascular imaging, axial non-enhanced CT or MRI – particularly with FLAIR sequence – are helpful in placing the craniotomy around the area of hemorrhage. The craniotomy should be more generous than one would otherwise fashion for a small nidus as identification of vascular landmarks, such as superficial veins, are very helpful in localization of micro-AVMs as illustrated in the accompanying case. After the craniotomy is elevated, the dura is opened under microscopic magnification to avoid tearing of the underlying neurovascular structures. Given the preexisting bleeding, hemosiderin staining of the subarachnoid space and adhesions of the arachnoid to the overlying dura are not uncommon.

Attention is then turned towards localizing the micro-nidus of the micro-AVM, which is often challenging given its small size. The surgical strategy we utilize is to identify the portion of the arterialized cortical draining vein which lies on the surface with intraoperative indocyanine green (ICG) angiography [7]. Often, the superficial portion of the cortical draining vein of the micro-AVM can be distinguished from uninvolved veins as its color is “reddish” due to its arterialization. Once identified, the superficial draining vein is followed in a retrograde fashion to the micro-nidus. In the case presented, the main draining vein of the micro-AVM was not readily identified on the initial intraoperative ICG as there was a thick layer of arachnoid that obscured it from our view. Once the arachnoid was carefully dissected, repeat ICG elucidated the position of the arterialized draining vein. Notably, in the illustrative case, only the distal half of the arterialized cortical draining vein filled in the early arterial phase. This helped in identifying the point of convergence between the deeper portion of the draining vein (within the sulcus) and the superficial portion of the draining vein which eventually drained into the superior sagittal sinus. Following the draining vein in a retrograde fashion leads to the micro-nidus. Once the micro-nidus has been identified, the hemorrhage cavity is entered, and the residual blood is evacuated. The small, arterial feeders supplying the AVM are then isolated and coagulated. Eventually, the devascularized micro-nidus is circumferentially dissected and separated from the hemosiderin stained pial surface bordering the hematoma cavity on one side of the micro-nidus. It is not uncommon that surgical exploration after removal of the residual hemorrhage identifies a thrombosed portion of the micro-AVM. After resection of the micro-AVM, hemostasis is obtained, and the resection cavity is carefully inspected for residual micro-nidus. ICG angiography is obtained which should demonstrate a lack of venous filling in the arterial phase and the area of the resected micro-AVM should appear as a dark spot.

#### Illustrative case

**Fig. 1:**
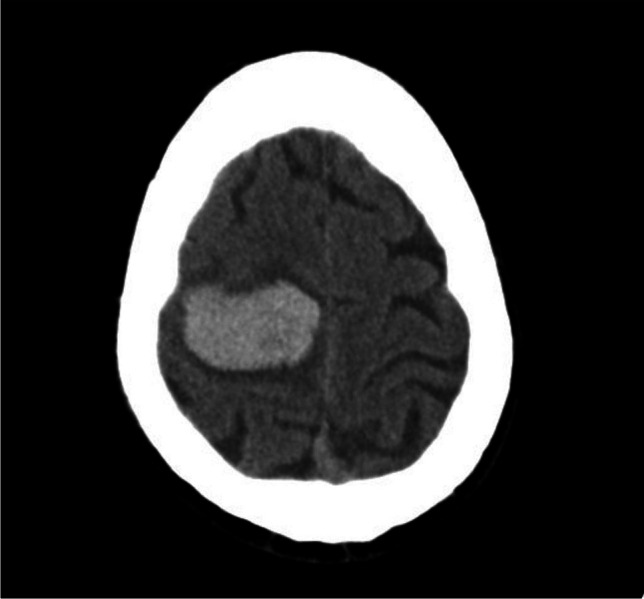
Axial non-contrast head CT demonstrating intraparenchymal hemorrhage in the posterosuperior aspect of the right frontal lobe

**Fig. 2:**
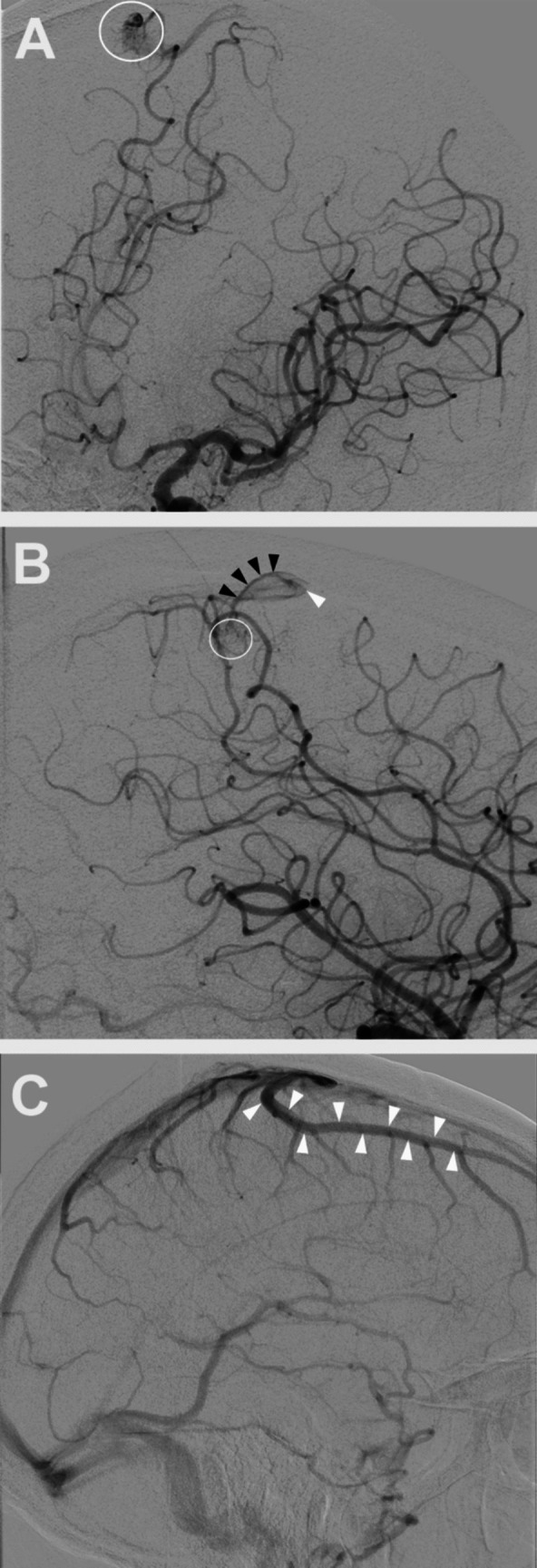
Preoperative catheter angiography. **A-** Oblique view of the selective left internal carotid artery injection demonstrating the parasagittal micro-nidus of the micro-AVM (white circle). **B-** Lateral view of the arterial phase of the selective right internal carotid artery injection demonstrating the area of the micro-AVM’s micro-nidus (white circle), the cortical draining vein into which the micro-AVM drains immediately anterior to the nidus (black arrow heads), and the location into which the cortical draining vein enters the superior sagittal sinus (white arrowhead). **C-** Lateral view of the venous phase of the selective right internal carotid artery injection demonstrating a large frontal vein coursing posteriorly to enter the superior sagittal sinus. As the vein enters the superior sagittal sinus immediately anterior to the micro-nidus of the micro-AVM, it is used as an intraoperative landmark to aid in identifying the micro-nidus

**Fig. 3:**
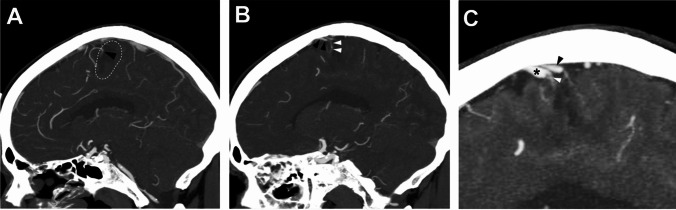
Preoperative stereotactic CT angiography. **A-** Sagittal view demonstrating the ill-defined micro-nidus of the micro-AVM (black arrowhead) at the anterosuperior border of the intraparenchymal hemorrhage cavity (white dashed outline). **B-** The small cortical vein into which the micro-AVM drains (white arrow heads) approaching the superior sagittal sinus immediately posterior to the large frontal vein (black arrow heads) identified on the preoperative catheter angiogram. **C-** The shadow of the small draining vein (white arrow) seen approaching the superior sagittal sinus between the large anterior frontal vein (black asterisk) and another posterior frontal vein (black arrowhead)

**Fig. 4:**
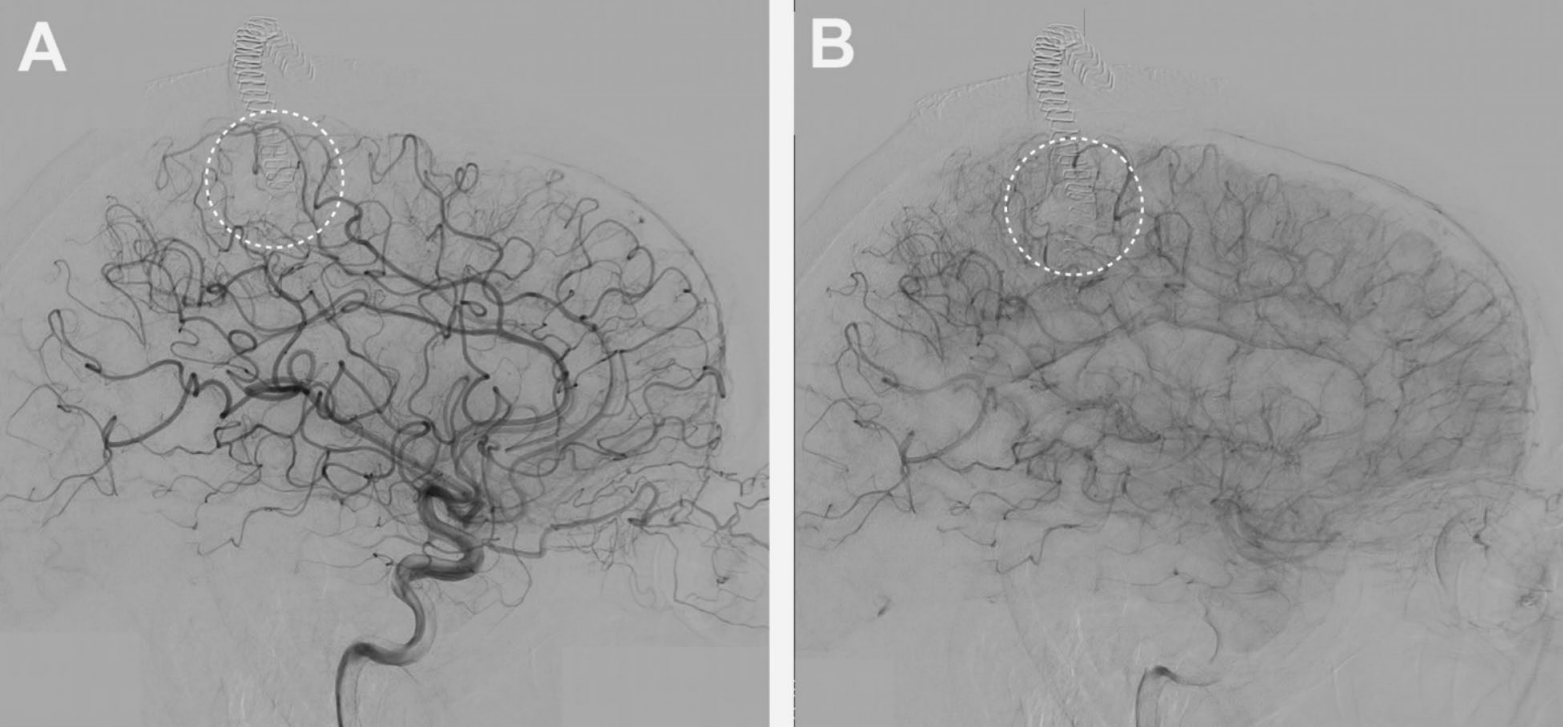
Lateral views selective right internal carotid artery injections arterial **(A)** and capillary **(B)** phases showing obliteration of the micro-AVM. The white circles depict the area of the micro-AVM prior to microsurgical obliteration

A 68-year-old man presented to an outside institution with sudden onset of headache and left-sided hemiparesis. CT scan at the time of presentation showed a large right posterior frontal intraparenchymal hemorrhage **(**Fig. 1**)**. Non-invasive CT angiogram did not show an obvious associated vascular lesion. He was initially managed medically, and, after an extended stay in inpatient rehabilitation, he recovered to an independent state with persistent left lower extremity weakness. At 6-month follow-up, catheter angiography demonstrated a right posterior frontal micro-AVM **(**Fig. 2**)**. He underwent microsurgical resection of the micro-AVM with the aid of pre-operative CT angiography **(Vid.1 &** Fig. 3**)**. Postoperative catheter angiography demonstrated complete obliteration of the micro-AVM **(**Fig. 4**)**.

### Indications

As most micro-AVMs present after hemorrhage, obliterative treatment is often indicated for most patients [8]. When treatment is indicated, options for micro-AVMs include open surgery, endovascular embolization, and radiosurgery [5]. Surgery is indicated for micro-AVMs that are superficially located in the cortical or subcortical spaces. Conversely, for micro-AVMs in eloquent or deep-seated locations, endovascular embolization is a reasonable alternative provided the micro-feeders can be catheterized close to the micro-nidus [5, 2, 6]. This avoids the risk of unintentionally occluding arteries feeding the surrounding brain. Stereotactic radiosurgery can be challenging for micro-AVMs as it is often difficult to identify a discreet micro-nidus to target. We utilize stereotactic radiosurgery for patients with incidental or deep seated micro-AVMs [3]. Regarding timing of intervention, when patients present with hemorrhage and an angiographic study does not elucidate the micro-AVM, some surgeons suggest immediate intervention to remove the hematoma and explore the hemorrhage cavity, while others suggest allowing for resolution of the hematoma followed by repeat imaging studies [1, 4]. As the hemorrhage secondary to the micro-AVM is rarely life threatening, in the presence of hemorrhage we recommend intervention in the subacute phase, as long as the angiogram does not show dangerous venous varices, pseudoaneurysms, or significant venous outflow obstruction.

### Limitations

Surgical localization of a micro-AVM can be challenging. Strategies to aid in localization of the micro-nidus include identifying precise adjacent anatomical landmarks, carefully inspecting the perimeter of the hemorrhage cavity as the micro-AVM resides there, and, when available, neuro-navigation and intraoperative ICG.

### How to avoid complications

Safely resecting micro-AVMs requires early identification of the main draining vein associated with the lesion. Additionally, the adjacent veins that are not associated with the lesion should be identified and protected. As the draining vein is followed deep into the sulcus where the micro-nidus of the micro-AVM is suspected to be, care should be taken to preserve the draining vein and minimize the risk of micro-AVM rupture [4]. When the micro-nidus and gliotic brain surrounding it are fully exposed, circumferential dissection begins, and care is taken to stay just superficial in the subpial plane to avoid injury to the adjacent parenchyma. ICG angiography at the end of the resection should be used to confirm obliteration of the micro-AVM. Further, though more of a consideration for AVMs that have previously hemorrhaged, dural reflection should be performed under microscopic visualization to sharply disconnect any adhesions between the dura and the often-thickened arachnoid.

### Specific information to give to the patient about surgery and potential risks

Patients should be informed about the rationale for treatment and existing alternatives. Potential risks of surgical resection include postoperative hemorrhage of the AVM leading to stroke, venous infarct, and other standard surgical risks.

## Ten key points

1. Micro-AVMs, as originally described by Yasargil, are AVMs with a nidus smaller than 1 cm.
2. Most micro-AVMs are located in the supratentorial space.
3. The majority of micro-AVMs are diagnosed after intraparenchymal hemorrhage.
4. Catheter angiography is the gold standard for correct identification of the Micro-AVM and understanding of its angioarchitecture.
5. Initial catheter angiogram might not disclose an arteriovenous shunt immediately after a large hemorrhage. However, repeat catheter angiography in 6–12 weeks is often diagnostic when there is a true micro-AVM.
6. Management strategies for micro-AVMs include observation, microsurgical resection, endovascular embolization, and stereotactic radiosurgery.
7. The craniotomy should be performed with the aid of neuro-navigation when possible, and it should be larger than one would typically fashion for a lesion this small as identification of surrounding vascular landmarks is often very useful in localizing the micro-nidus.
8. Intraoperative ICG angiography is useful to identify the superficial cortical draining vein that is arterialized by the micro-AVM. This vein can then be followed in a retrograde fashion to identify the micro-nidus.
9. In the setting of hemorrhaged micro-AVMs, the micro-nidus consistently is located at the periphery of the hemorrhage cavity.
10. Complete obliteration of the micro-AVM can be confirmed with ICG angiography showing lack of venous filling in the arterial phase and the resection bed of the micro-AVM appearing as a black dot.

## Electronic supplementary material

Below is the link to the electronic supplementary material.

ESM 1 (MP4 367 MB)

## Data Availability

No datasets were generated or analysed during the current study.
